# Why here? Factors influencing Palestinian refugees from Syria in choosing Germany or Sweden as asylum destinations

**DOI:** 10.1186/s40878-018-0094-2

**Published:** 2018-10-15

**Authors:** Jason Tucker

**Affiliations:** Malmö Institute for Studies of Migration, Diversity and Welfare (MIM), Malmö University, Malmö, Sweden

**Keywords:** Asylum destination, Citizenship, Germany, Sweden, Stateless refugees, Palestinian refugees from Syria

## Abstract

This paper presents the findings of 33 interviews, carried out in 2017, examining the factors influencing Palestinian refugees from Syria (PRS) in choosing Germany or Sweden as asylum destinations. The findings showed that there was a very high degree of destination specificity towards Sweden for nearly all of the participants. This was based on their desire to reach Sweden due to its accessible citizenship as compared to other European or Arab states. This paper details how most of the refugees had conducted research, drawing on information from social networks and other sources, in order to establish in which European country they could most easily and quickly acquire citizenship. As a consequence of the prioritisation of resolving their and their families’ statelessness as quickly as possible, the interviewees often devalued social and human capital. Considerations related to economic or educational opportunities played only a marginal role in the decision making. This research finds that in order to better understand the migration of stateless refugees, their desire to resolve their statelessness should be considered as a potentially significant aspect of their choice of asylum destination.

## Introduction

Statelessness, or not having citizenship of any state, is beginning to be recognised as an area of interest in forced migration studies. In 2016, the Office of the United Nations High Commissioner for Refugees (UNHCR) reported that there were 22.5 million refugees globally, of which at least 6.6 million were stateless (Institute on Statelessness and Inclusion [ISI], 2014; UNHCR, 2017). The lack of a legal bond of citizenship to a state, as faced by stateless refugees, should not be confused with the loss of ‘effective’ citizenship. Non-stateless refugees lack effective citizenship. Indeed, their need for international protection stems, in part, from this. For stateless refugees, it is not a case of ‘effective’ or ‘ineffective’ citizenship as legally and or factually they have no citizenship.

Despite a quarter of all refugees being stateless, research on the impact of statelessness on refugees (or indeed refugee-ness on the stateless) is notably scarce. This research sought to address one gap in our understanding by questioning if statelessness impacts the asylum destination decision making of refugees. To achieve this, 33 in-depth interviews with stateless refugees in Germany and Sweden were carried out during 2017. Given that neither Germany nor Sweden, as with most states globally, has law or policy in place to comprehensively, accurately or transparently identify the statelessness of refugees, the research focused on Palestinian refugees from Syria (PRS). The statelessness of this sub-group is, by and large, recognised by both Germany and Sweden. The same cannot be said for other stateless refugees in the two countries.

The interviews focused on why the participants decided upon Germany or Sweden as the country in which to seek asylum. The findings showed that there was a very high degree of destination specificity towards Sweden for nearly all of the participants. This was based on their desire to reach Sweden due to its accessible citizenship as compared to other European or Arab states. Thus, many of the participants prioritised being able to resolve their, and their families’ statelessness, as quickly as possible. Most of the participants had conducted research before leaving Syria, drawing on information from social networks and other sources, to establish in which European country they could acquire citizenship.

Other factors were also raised by the participants as influencing their destination decision making. These included being able to acquire a status that would allow them to access citizenship, joining family members and limited choice. Economic and educational opportunities or smugglers and agents, played only a minor role. Interestingly, social and human capital were often devalued, compared to speedy acquisition of citizenship. In summary, the research finds that in order to better understand the migration decisions of stateless refugees, their statelessness and their desire to resolve this should be considered.

## Research background

### Palestinian refugees from Syria

While around half a million Palestinian refugees resided in Syria before the current conflict, it was estimated that by 2017 over 120,000 of them had fled the country (The United Nations Relief and Works Agency for Palestine Refugees in the Near East [UNRWA], 2017a). This exodus is unsurprising given that Palestinians have been targeted by various state and non-state actors in Syria ("Palestinians of Syria," 2017).

The Palestinians have been framed as in a unique situation by international law. This led to their exclusion from protection under the refugee and statelessness conventions. This applies if they are in the areas of operation of UNRWA: These areas being Lebanon, Syria, Jordan, the West Bank and Gaza. The secondary movement of Palestinian refugees from Syria to other states where UNRWA operates has not been a smooth transition, with many PRS falling into a ‘protection gap’ between the mandates of UNHCR and UNRWA (Erekat, 2014). As such, Syria’s neighbouring states, where most of the PRS can be found (see UNRWA, 2017a), provide little in terms of relief or protection for the population. In Lebanon for example, almost 90% of the PRS live below the poverty line (UNRWA, 2017b), while in Jordan basic services are denied to the population and they face deportation to Syria (UNRWA, 2017c).

Due to these and other factors, onward mobility is steady, with estimates that between 2011 and 2016 nearly 85,000 PRS sought asylum in Europe ("Palestinians of Syria," 2017). In 2015 and 2016, refugees from Syria constituted the largest group of asylum seekers to Europe (Eurostat, 2017a). Germany and Sweden have been, and continue to be, very popular asylum countries in Europe. The figures reflect this, with the two countries receiving the majority of asylum applications between 2013 and 2016 (Eurostat, 2017b).

Due to the lack of data on those identified as stateless, it is not possible to know for sure how many of these applications were from PRS. It has been reported that from 2012 to 2016, 698 PRS were granted asylum in Germany, though no information on pending decisions or the granting of other protection statuses is available ("Palestinian Refugees Multiple Displacement," 2017, p. 25). While no specific information on the PRS in Sweden is collected, between 2012 and 2016, 26,1268 asylum applications from those classified as stateless were recorded (Statistics Sweden, 2017). Thousands more were recorded as having unknown nationality, a result of the lack of law or policy for the Swedish Migration Agency (*Migrationsverket*) to draw on in establishing the statelessness of refugees (Tucker, 2017). The Swedish and German migration authorities can draw on institutional memory here, as there is a history of Palestinian refugee/migratory flows to the two countries. Both host sizable Palestinian diaspora who arrived from Lebanon during the civil war in the mid 1970s to the 1990s and in waves from Iraq between 1990 to today (Doraï, 2003; Fiddian-Qasmiyeh, 2016).

It could be read that the PRS are just a narrow reflection of a wider migration trend to Germany or Sweden. The available data, while limited, would suggest that this may not be the case. In 2015 and 2017 Germany received by far the largest amount of asylum seekers from Syria (Eurostat, 2018). However, if we look at the period from 2011 to 2017 one can see a significant amount of variation and spread in the European asylum destinations of asylum seekers from Syria (Eurostat, 2018).

The data on the destination countries of PRS in Europe is limited. When they are identified they are normally classified as stateless, or a variation thereof, with Syria as their country of origin. Therefore, we can only turn to the available data on *all* stateless asylum seekers. This shows that during the same period there was significantly less variation in the destinations of this sub group of asylum seekers. Sweden received the majority of the stateless asylum seekers in Europe during the period 2011 to 2017 (Eurostat, 2018). States who have a history of receiving and recognising certain groups of stateless asylum seekers, such as The Netherlands, France and Denmark, also received steady numbers which were proportionate to the overall asylum numbers throughout the period (Eurostat, 2018). Thus, stateless asylum seekers seem to follow different migration trajectories than non-stateless asylum seekers in Europe. This is an area of enquiry that requires further research as it would be interesting to compare the movement of stateless and non-stateless asylum seekers from the same country of origin.

### Factors affecting asylum destinations

When considering the literature on asylum destinations, what can be described as the classical view claimed that, being forced to flee, asylum seekers and refugees had little agency in their choice of destination (Böcker & Havinga, 1997). This position has been the subject of criticism, with the role of agency and choice being recognised in this decision-making process (Zolberg, Suhrke, & Agyayo, 1989).

More recently, country specific studies, such as that of Brekke and Aarset (2009) for Norway, or Robinson and Segrott (2002) for the United Kingdom, have proved enlightening, highlighting that choice of destination may change for refugees during their migration, as well as developing a hierarchy of reasoning for their decision making. Commentators have also utilised comparisons between those from different countries of origin in destination states. Day and White's (2002) research on the United Kingdom showed that in understanding the destination choices of Bosnian and Somali refugees one must not only consider individual choice but also institutional explanations in understanding the patterns of asylum destinations. Social networks, information exchange and chain migration have also been shown to influence destination decisions (Koser & Pinkerton, 2002), as have trans-border ethnic linkages (Rüegger & Bohnet, 2015). Other considerations include the image or perception of the destination country, language, labour market opportunities, education, welfare and the desire to live in a state with democracy, freedom and human rights (Brekke & Aarset, 2009; Neumayer, 2004).

However, research on stateless refugees, specifically considering how their statelessness impacts their choice of destination country, has, to the best of my knowledge, not been undertaken. As such, this research aims to begin to address this gap, unpacking the role of statelessness in the decision-making process. Such research not only allows us to address this gap in our knowledge, facilitating a more nuanced understanding of migration, statelessness and refugee-ness, but it also serves to inform policy related to refugee flows. This is because, as will be discussed below, for those interviewed, their statelessness and their desire to resolve this, played a significant role in their asylum destination decision. Thus, in trying to understand and/or manage migration flows at a national or regional level, one must recognise that variations in access to citizenship could be a significant factor in stateless refugees’ onward movement and choice of asylum destination.

### Stateless refugees

The study of statelessness originated in the legal field, initially focusing on the analysis of nationality law and later framing statelessness as a human rights question (Manly & van Waas, 2014). Whereas citizenship, which is often seen as the antithesis of statelessness, is described by Joppke (2010, p. 1) as “a notoriously polyvalent concept”, the same cannot be said for statelessness, which has yet to break free from its legal origins.

Previous research has focused mainly on in situ stateless populations, or the situation of non-refugee stateless migrants. Some scholars have however started to recognise that the statelessness of refugees’ is both meaningful and impactful on refugees’ lived experience, or that refugee-ness is impactful on the lives of the stateless. We can see examples of where commentators have explored statelessness as a cause or consequence of forced migration (ISI, 2014), as a barrier to securing solutions for refugees (Hovil, 2016; Sen, 1999; Sen, 2000), statelessness and refugee protection (Darling, 2009), persecution (Fullerton, 2005) and deprivation of nationality (Fripp, 2016).

Yet, despite some developments, it continues to be researched in distinct national legal contexts, with statelessness itself being seen more as a anomalous legal status/puzzle than a multifaceted lived experience that shapes how people move between, within and through these local, national and regional legal contexts. For example, even very comprehensive texts on refugee law and policy that are prominent in refugee studies have only a few fleeting references or footnotes to statelessness (see Feller, Türk, & Nicholson, 2003; Goodwin-Gill & McAdam, 2007; Hathaway, 2005; Hathaway & Foster, 2014).

## Methods

Understanding why stateless refugees sought asylum in certain places is by no means an easy task. However, by drawing on the narratives, explanations and perspectives of participants, we can begin to unpack the decision-making process of this under researched group. For the purposes of this analysis, the definition of a stateless person builds upon that of the 1954 Convention on the Status of Stateless Persons, to which both Germany and Sweden are state parties, namely, “... a person who is not considered as a national by any State under the operation of its law” (United Nations General Assembly, 1954, Art. 1.1). All but one of the participants had been classified as ‘refugees’ in Germany or Sweden, though they held different residency statuses. One woman was on a student visa living with her husband who had refugee status.

Palestinians constitute by far the largest stateless population globally, as well as being one of the largest refugee populations (ISI, 2014). As such, they provide a useful point of departure to explore statelessness and refugee-ness. Palestinian refugees can and often do self-identify as Palestinian/stateless, with many being able to prove this with documentary evidence. In addition, the authorities in Germany and Sweden know of and recognise Palestinians as stateless. Therefore, in terms of beginning to understand the impact of statelessness on refugees they are one of the few ‘identified’ stateless refugee populations, making them distinguishable from non-stateless refugees.

Diversity, in terms of age, sex, family situation, socio-economic background and migration history was sought when participants were selected. Eleven participants were interviewed in Germany (four women and seven men), in nine interview sessions (one interview was with a married couple and another was with an aunt and her nephew). Twenty-two people were interviewed in Sweden (seven women and fifteen men) during eighteen interview sessions (two were held with married couples, one with a mother and son and another was with two friends). The ages of those interviewed ranging from nineteen to seventy one years old. The interviews took place between February and December 2017.

The vast majority of the interviews were undertaken by the Palestinian League for Human Rights-Syria, as part of their research on the protection gaps faced by PRS. Only two were undertaken by myself as part of another ongoing project.^1^ With regard to my involvement in the data collection process, I acted as an advisor to the Palestinian League for Human Rights-Syria research project. I worked closely with the researcher conducting the interviews before, during and after the data collection. This included determining the research questions and adapting the research as new themes emerged that required further exploration. Partnering with the Palestinian League for Human Rights-Syria meant that their well-developed network could be utilised in a snowball sampling technique. To ensure confidentiality, pseudonyms have been used and any information that would allow the participants to be identified has not been published.

Given that the research was exploratory, semi-structured in-depth interviews were chosen as the method of data collection. This meant that while some pre-determined broad themes could be focused on, there was significant scope for the participants to engage in open discussion using narratives and stories (DiCicco-Bloom & Crabtree, 2006). Narratives were central in the data collection, with participants being asked to describe their lived experience of Palestinian-ness/statelessness before they left Syria, through to their life in the destination country. While this often resulted in their reasons for choosing one country over another being raised, similar to the work of Brekke and Aarset (2009) in understanding Norway as an asylum destination, participants were asked ‘Why Sweden/Germany?’ to draw out more explicit responses. Care was taken not to lead the participants’ responses into preconceived notions of their decision-making process. Here the open ended narrative format proved very useful. For the majority of the interviews translators were used as the participants wished for it to take place in Arabic, though several interviews were conducted in English.

A comparative analysis of one stateless group in two destination countries was decided upon for this research for several reasons. First, gaining a breadth of understanding by looking at different national contexts was important as research on stateless refugees’ asylum destination decisions had yet to be undertaken. This exploratory research therefore sought to begin to unpack if and how statelessness impacted asylum destination decision making, then to compare this to the already developed body of literature on refugees’ decision-making in general. Second, the comparative approach meant that this understanding would not be limited within a specific national context (Green, 1994). Third, Germany and Sweden make for interesting and highly relevant contexts given the large number of refugees they receive in comparison to other European states and Sweden’s relatively liberal access to citizenship compared to Germany’s much more restrictive naturalisation regulations.

There were limitations to the research that should be made explicit. First, the sample was small, especially in the case of Germany. The larger number of participants in Sweden was due to the ease at which they could be identified and accessed. It may also relate to the larger numbers of those of interest to this research in Sweden as compared to Germany - though this cannot be stated with certainty given the lack of data. Geography played a role here as well. In Sweden refugees are more geographically concentrated in or around the four largest cities. In Germany identifying participants proved more challenging due their geographic dispersal and fewer connections on which the researchers could draw. In addition to this, given the focus of the research is on Germany and Sweden, the research only includes those who have ‘made it’, or in several cases, nearly made it.

## Why here? Unpacking the asylum destination decisions

### The search for citizenship

How the participants conceptualised their statelessness, and how this changed over time, should be considered, as this allows for a more in-depth understanding of their desire to acquire citizenship. When the participants reflected on their life in Syria before the conflict, all of them noted that they saw little difference in their treatment as compared to Syrian citizens. Several claimed that, while recognising that they did not have a ‘homeland’, they did not feel ‘stateless’ in Syria. The only difference they could recall was their lack of voting rights. However, there was a clear distinction drawn between their situation pre-and post 2011. After 2011 when the Palestinians began to be targeted by state and non-state actors, the participants recalled how they felt ‘stateless’ or ‘not Syrian’ for the first time. Before the war most noted that they had never considered leaving the country to try to secure citizenship elsewhere. After 2011 they realised that they had only been temporarily tolerated in Syria and had to try and find a more permanent solution to their refugee-ness and statelessness elsewhere: the search for citizenship began.

When asked the question ‘Why Sweden?’, nearly all the participants in the country stated that it was the possibility of being able to quickly and easily resolve their statelessness through the acquisition of Swedish citizenship that was the most influential pull factor. Until the introduction of the temporary asylum laws in 2016^2^, Sweden had very liberal naturalisation criteria and granted permanent residence to refugees as well as those granted subsidiary or humanitarian protection (Brochmann & Seland, 2010; Tucker, 2017). Refugees only had to reside in the country for 4 years to apply for citizenship and there were no naturalisation criteria based on language proficiency, passing citizenship tests, integration assessments or proving self-sufficiency for refugees to meet (Migrationsverket, n.d.). While in other states refugees can acquire citizenship in a shorter timeframe, for example with only 3 years residence in Greece or Hungary (GLOBALCIT, 2017), to benefit from facilitated access to citizenship, one must be recognised as a refugee. Recognition rates, as well as the granting of refugee status, rather than subsidiary or humanitarian protection, are very low in these states (Eurostat, 2017c).

For many participants, this decision to head for Sweden was made when they were in Syria. As such they may be understood as being in an “anticipatory refugee scenario” (Kunz, 1973). This is because they had time to research destination countries, draw on social networks and secure the required resources before they fled. As was the case of Ahmed in Sweden:

*“I read a lot about the options I had and I asked people I knew that were living here [Sweden]. People gave me good feedback in terms of the life here, the treatment of refugees and migrants, that kind of thing. That’s how I made my decision.... My first criteria was getting permanent residency that would eventually get me citizenship and a passport that allowed me to move around freely.”*

A few cases however could be understood as being in an “acute refugee scenario” (Kunz, 1973): fleeing without having time to prepare. Those in this situation claimed that the fear of being ‘put on the list’ of the authorities, was the tipping point that lead to their unplanned/earlier than expected flight. As Ghalia, a medical worker from Yarmouk, recalled when she was trying to enter the camp to go to work:

*“I was at a checkpoint near the camp and one of the Shabeeha [demeaning name for pro Assad militants] called out my name. He said to me, ‘You’re a doctor and you’re trying to go into the camp to help the Free Army.’ I was afraid that my name had become on a list, so I never tried again”.*

In these cases, it was only after they had fled that the decision was made to try to reach Sweden. After being arbitrarily detained in Greece due to his statelessness, Ayham recalls the impact of his statelessness on his decision making:

*“All my decisions since that day [of leaving the detention centre] — and even before — were taken with one issue in mind: what’s the fastest way to get a citizenship and how can I get a passport? In Sweden or anywhere else. I wanted to get citizenship.”*

The importance of citizenship for some of the participants was highlighted when they noted that in choosing Sweden they were sacrificing the possibility of better utilising human or social capital in other countries.

*“The most important thing here is, why Sweden? Why was Sweden my final destination? Because it’s the fastest country to give citizenship and a passport. So, for example, why didn’t I go to England? I speak English and it’s a good country... [B]ut why did I chose Sweden? Because in England it’s incredibly difficult to get citizenship compared with Sweden.” (Mohammed, Sweden).*

*“The environment in Austria and the work opportunities in Germany are a lot better than here. We came here [Sweden] so that we could get citizenship and so that we could travel.” (Ahmed, Sweden).*

Several participants noted that resolving *their* statelessness was not their only concern, but by using family reunification they would also be able to bring their family members over to resolve their statelessness as well.

*“For me citizenship was the most important thing. If I don’t get that, then I couldn’t bring my son out and then he could get citizenship.” (Ayham, Sweden).*

Only one participant, Ghalia, claimed that while Sweden was her first choice, she would also have been willing to seek asylum in Germany.

*“Four of us took a car, with the driver it was five, just driving directly from Italy to Sweden. It wouldn’t have had a problem if we’d been caught in Germany and had to stay there, it made no difference to me.”*

In comparison to Sweden, Germany has much more restrictive naturalisation criteria. This includes 8 years residency in the country (with exceptions), language proficiency, passing citizenship tests and proving self-sufficiently for oneself and ones’ family (Asylum Information Database, n.d.). Half the participants in Germany, who intended to reach Sweden, Norway or The Netherlands, described access to citizenship as one of the main factors in choosing their intended destination. Tasneem, whose father was stopped in Germany and joined him through family reunification, recalls her families’ reasoning in trying to get one member to Sweden:

*“He [my father] didn’t choose Germany. He wanted to go to Sweden... — you know, Sweden was famous for Palestinian people from Syria... On the way to Sweden, the police caught him in Nuremberg in the south of Germany and he couldn’t go on. They took all the money he had and he had no money to travel to Sweden.”*

Tasneem goes on to explain why Sweden was ‘famous’ for PRS:

*“Sweden was famous for the citizenship thing: for example, underage Palestinian Syrians [those under eighteen years old] could get citizenship after two years, and over-age would get it after five or six years.”*

Of the remaining participants in Germany, who were not aiming to reach Sweden, Norway or The Netherlands, while they did not state that citizenship was influential in their decision making, all noted that acquiring permanent residence in Germany was a very significant factor. This should not be read that citizenship was seen as unimportant to these participants. Rather it was just not prioritised over other factors, as is apparent when Sami, who travelled with his wife Deema to Germany, discussed his decision-making process:

*“I have a brother who has been in Germany for a long time, and he has German nationality. I also have some uncles and other relatives in Germany. But my first intention was to either go to Holland or Sweden because as far as I’m aware, it’s easier and faster for Palestinian-Syrians to get nationality. But the thing that made me stay in Germany was the fact that [Deema] had that visa to come to Germany.”*

Here we can see the devaluation of social networks, that of his brother and other relatives in Germany, as compared to acquiring citizenship as quickly as possible in Sweden. It was only due to Deema having a visa to study in Germany that Sami decided to make it the country where he sought asylum.

Finally, when asked what acquiring a citizenship would mean to them, the ability to travel was highlighted as central by all participants in both countries. ‘Travel’ referred to being able to return to Palestine (for short visits), to visit family and friends, to move for work and to return to live in Syria after the war. On their return to Syria, the participants claimed that as stateless persons they would not be allowed to go back, but if they had a European citizenship it may be an option. The role of citizenship in their return also related to their appreciation of the temporariness of their rights in Syria and the need for a back-up plan if they were to return. As Jamal explains:

“*After we get citizenship [in Sweden] and things got better in Syria, maybe we’d go back. But we need citizenship to be able to travel — in case things kick off again.*”

### Family and social networks

As has been shown in other research on refugees’ asylum destinations, (see for example Koser & Pinkerton, 2002), as well as work on chain migration more generally, social networks and the information exchange through these proved influential in the destination decision making of the participants. The findings on the role of social networks can be broken down into two aspects in this research. The first being where the participants arrived through formal family reunification procedures. The second is when social networks were drawn on for information about possible destination countries, and/or to provide participants with the necessary resources to try and undertake the journey.

A common theme for all those interviewed was their knowledge of which countries in Europe accepted and recognised PRS as refugees, as well as the status they would receive. Some participants were even provided with information from social networks on which documents to bring from Syria, as well as how and when to raise their statelessness with certain authorities on their way to, and when arriving in, the destination country. While the perception of the overall asylum system, based on information from social networks as well as other sources, was shown to be influential, for those interviewed this decision was based on these countries treatment of refugees similar to them, i.e. PRS. As such, it was not just the residency and citizenship laws and policies that were influential. It was also information about their likelihood as PRS to secure a legal status that would in turn allow them to access citizenship.

Of those participants in Sweden, or originally intending to end up there, all referred to family or social networks as a significant factor which was intertwined with the acquisition of citizenship in their decision making. As comments from Dawoud and Mamdouh reflect:

*“And why Sweden? First of all, my siblings are here in Sweden. And more importantly, Sweden would give me the right to nationality. Citizenship.” (Dawoud, Sweden).*

*“I already had relatives here; a sister-in-law and a sister that had lived in Sweden for a while. There wasn’t a particular reason, I mean … although it’s easier to get papers here, to get citizenship and a passport.” (Mamdouh, Sweden).*

However, those without family in Sweden all had close family elsewhere, either in Europe or in Arab states (normally both). While several participants acknowledged that it would have been better if they could have sought asylum in a place where they had close family ties to draw on, these concerns were outweighed by the prospect of acquiring citizenship.

### Economic or educational opportunities

The pull factors of perceived economic and educational opportunities in Germany and Sweden rarely featured as being influential in the participants’ asylum destination decision making process. Only one participant in Sweden noted that their choice was based, in part at least, on such opportunities, though social networks and the likelihood of them being granted asylum was also influential.

*“I didn’t have much time to make the decision or to plan, but I did some research online about where the best place was [to seek asylum]. I already had some friends who’d come to Sweden as well as an aunt living in Sweden, so through asking them and getting recommendations and thinking about my family — back then I had one child and now I have two — I prioritised Sweden because it meant a better future for my kids; in terms of education and so on. And also, for me and my wife, we are both highly educated... I thought it would be easier for us to find jobs as opposed to anywhere else. I thought that Sweden would be more suitable for us as a family — and at that time it was faster to get residency, it didn’t take much time relatively speaking — but also because of the economic situation.” (Mansur, Sweden).*

Imad in Germany was the only participant to explicitly claim that economic opportunities were more important than considerations related to securing residency or citizenship. Imad already had a brother in Germany and he wanted to bring his wife (who was in Lebanon) over to Europe as soon as possible. He was also responsible for supporting his parents and siblings who were still in Syria.

Imad: *“There were work opportunities here [in Germany] and the economy is strong. And it’s a great country.”*

Interviewer: *“So for you, is work more important than legal things related to residency or citizenship?”*

Imad: *“Yes, a lot more important.”*

Both Mansur and Imad made the decision to move to Sweden or Germany respectively before leaving Syria. However, for Hassan, the educational and economic opportunities of Germany were a pull factor after he realised that these were not available in his initial destination, Spain. At 20 years old, Hassan was the second youngest participant. He had travelled from Syria through Egypt, Spain and then to Germany with several of his brothers. The decision to move from Spain and seek asylum in Germany was undertaken in the knowledge that it would delay his possibility of acquiring a citizenship, as he explains:

Hassan: *“When I went to Spain, Spain was actually good. You could get the papers in a year or two years, but you’d get five-year asylum and then after those five years are up and you’re economically independent, you can get the nationality. So, the problem in Spain was that if I wanted to study, it’d be hard for me and I’d be on my own, and also work. It’s hard to find work there.”*

Interviewer: “*So getting nationality in Spain is quicker than in Germany?*”

Hassan: “*Yes, quicker.*”

Interviewer: “*So why did you decide to come to Germany?*”

Hassan: “*Because I noticed that I wouldn’t be able to study, because I wouldn’t have enough money, and also because if I wanted to get a job there [in Spain] then it’d be hard.*”

### Limited choice in where to seek asylum

To understand the limitations of where PRS can seek asylum, one has to consider the situation the PRS face in Syria’s neighbouring states as well as the wider region. While there is not space here to detail variations in these national contexts, other researchers have summarised this as stemming from what is referred to as a ‘protection gap’. As Sayigh (2013) explains:

This differentiation between Syrian and Palestinian refugees on the part of neighbouring Arab states mirrors a differentiation at the level of the United Nations. UNHCR’s mission specifically excludes Palestinians. Instead they are served by a separate agency, UNRWA, and UNRWA’s mission does not include protection. Thus, even though the UNHCR and UNRWA are working jointly under the Syrian Regional Response Plan to assist all refugees from Syria, it is not clear so far if the UNHCR will exercise its protective mission to pressure Jordan and Lebanon to stop turning away Palestinians seeking sanctuary, or to prevent discriminatory treatment.

For the PRS in Jordan and Lebanon there are no legal mechanisms to regulate their entry or stay, leaving them in limbo, as well as the population being the target of state and societal discrimination (Eshnaiwer, 2015; Human Rights Watch, 2014; Sayigh, 2013). This includes fees specifically for PRS to renew visas, restricted movement, limited access to services on par with Syrian refugees and instances of forced return to Syria (Sayigh, 2013). The PRS even face differential treatment by UNRWA compared to other Palestinians, with the organisation “register[ing] them as non-registered assistance recipients to ensure that the PRS remain registered with UNRWA-Syria, but their basic needs are covered by UNRWA in the host country” (Eshnaiwer, 2015, p. 9). This protection gap for the PRS exists within contexts where stateless people in general face a complete lack of any real prospect of acquiring a legal status, citizenship, or formal employment (ISI & NRC, 2016).

For all participants in both Germany and Sweden, the difficulties PRS faced in Syria’s neighbouring states, and in some cases the wider region, impacted their decision to seek asylum in Europe. Tasneem and Ayham in Sweden reflected on how their statelessness limited their choice of possible destinations as compared to the opportunities available to refugees with Syrian citizenship:

*“Syrians have the choice to go to Lebanon; Syrians have the choice to go to Turkey, to Egypt and they can go to Egypt right now. To Jordan. Palestinians can’t go anywhere other than just being stuck in Syria.” (Tasneem, Sweden).*

*“If I was Syrian, I’d go to Britain, to Britain or Germany. But when I looked at my options, Sweden was the easiest and most secure way.” (Ayham, Sweden).*

Ali in Sweden reflected upon how if he had not been PRS he would have headed for the United Arab Emirates (UAE) to best utilise the social networks and human capital he had, instead of Sweden where he had only weak social ties:

*“If I was Syrian, I’d probably just have gone to another Arab country — I have three brothers in the UAE but I couldn’t get a visa to get to them. Even to get a visa to Lebanon wasn’t an easy thing. Choosing to go through Turkey to Sweden was because I’m Palestinian and, because I’m Palestinian, I can’t get a visa to any Arab country, so I had to go that way. First, before deciding to go to Lebanon... I was trying to get a visa to the UAE but it didn’t work out. The UAE would have been a better choice for me; I had three brothers there, it’s an Arab country, so I could have started working again easily there.”*

Khalid explains why he undertook the dangerous and costly journey to Sweden from Lebanon. Given the lack of legal status for PRS and the protection gap, he was forced to live ‘underground’ and to enter Europe irregularly:

*“Of course. In the worst-case scenario, I could have stayed in any country and applied for asylum according to the UN’s quota system [resettlement programme] and waited. As a Palestinian I never had that opportunity because UNRWA is in charge of us. And in their current situation, it’s not their mission to deal with asylum or protection cases. A lot of the Palestinians in Lebanon — and Palestinian-Syrians in Lebanon — or in Turkey face these problems. They cannot register to seek asylum [through resettlement] to Canada or Australia or Europe or wherever, it’s just for Syrians.” (Khalid, Sweden).*

Due to the lack of legal pathways to seek asylum, Deema who travelled to and resided in Germany on a student visa, when asked ‘Why Germany?’, recalled:

*“… actually, the [university] course was just a way to get out of the country [Syria], it’s not like I wanted to come and study here.”*

With regard to the role of traffickers, smugglers and agents in choosing the destination country of asylum seekers to Europe, as highlighted by Böcker and Havinga (1997), this was only raised by two participants.

*“We didn’t choose it [Germany]. When we crossed the sea from Turkey to Greece, there they gave us a paper because our passports are not international — they didn’t even understand it. We had a translator, and he translated our passport and papers and stamped it; and he wrote on it that our final destination was Germany. And we weren’t able to travel more after that.” (Noor, Germany).*

*“The decision wasn’t certain in the beginning. The idea was just to make the journey and cross the sea, and then once we arrived to Italy we considered what country we would go to. I did some research before, like to see what the best country was to go to as a Palestinian-Syrian. The best ones were Sweden, Norway, The Netherlands and Germany. I didn’t think that Germany was a good choice. And we all knew that Sweden is good for Palestinians from Syria. In the end, the smuggler sort of made the choice for us because he told us that there was a bus direct from Italy to Malmö. So, we came to Sweden.” (Farid, Sweden).*

It should be noted that nearly all of the participants left Syria and entered Europe with the use of traffickers, smugglers and agents. This included using them to secure fraudulent documents, arrange flights/sea-crossing as well as transportation up through Europe. Therefore, there was significant scope for these actors to influence the final destination of the participants. Yet, with the exception of Noor, when participants arrived in a country other than their desired asylum destination, they sought to complete their journey.

## Discussion

In general, the findings from the interviews showed a very high degree of destination specificity towards Sweden for the vast majority of the participants. This was based on the participants’ perception of the ease and expediency of securing permanent residency and acquiring citizenship in the country.

While citizenship was specified as an important pull factor, this was often interrelated with other factors. Most notably, conceptualisations of the role of social networks prove useful to draw upon here to deepen our understanding of asylum destination decisions. Many of the participants had detailed knowledge of the asylum system and citizenship laws and policies in several European countries, having done research which drew on a range of sources. The most significant of these was information from social networks. This information related to the treatment of asylum seekers in the country, the likelihood of their asylum claim being accepted and the ease at which citizenship could be acquired thereafter for themselves and their families.

This corroborates previous work on the perception of the asylum system being influential in destination decision making (see Brekke & Aarset, 2009; Neumayer, 2004). However, it is argued here that for the participants the perception of the asylum system was just part of their overall focus on access to citizenship. This was because without being recognised and given a legal status the acquisition of citizenship would not be possible.

With regard to social networks, nearly all those interviewed had close family in countries other than Syria. In addition, all of them had drawn on information from social networks during their journey (though not always with regard to their asylum destination). Interestingly, about half the participants in Sweden noted that they had not drawn on strong family ties in other countries, explaining that these were rejected in order to reach Sweden to access citizenship as quickly as possible. This was illustrated in three cases where close family members living in Germany and Denmark drove the participants to Sweden to complete the final leg of their journey. The participants decided to decline offers to stay with these family members long term, instead deciding to continue to Sweden where they had no, or very weak ties, but where they knew they could acquire citizenship faster. In addition, many participants stayed with family members for short periods of time in various countries as they moved up through Europe. These were most often uncles, aunts and cousins, but in a few cases, they were siblings and parents. Building upon the work of Collyer (2005), we should consider that it is not just strict immigration policies that can impact migrants’ ability to draw on strong familial ties, but for stateless migrants it would be advisable to also consider the impact of citizenship law and policies in this regard.

In relation to this, we can also see the devaluing of human capital compared to acquiring citizenship. Several participants noted how they could have more effectively capitalised on human capital in countries other than Sweden (such as knowledge of languages etc.). However, this was either not possible due to restrictive immigration rules, or not as important as citizenship. Thus, while social networks and human capital were influential in asylum destination decision making this did not necessarily lead to chain migration as it was most often devalued as compared to acquiring citizenship.

The decision on which country to seek asylum in was made at various times by the participants. For the majority, it was made while still in Syria. A few participants however undertook onward migration, changing their asylum destination as a result of their statelessness posing an insurmountable barrier in securing a legal status, formal employment or a basic level of protection (with the exception of Hassan who moved due to the lack of educational and economic opportunities for refugees in Spain in general). Onward migration due to the participants’ statelessness occurred in Syria’s neighbouring states, as well as for those who had reached Europe.

While single group studies such as this allow for a more in-depth analysis of migrant experiences, they run the risk of neglecting experiences which are common to migrants outside of the group (Archdeacon, 1985; Vecoli, 1972). In terms of how far the PRS can be seen as ‘representative’ of stateless refugee more generally, the role of UNRWA and some Palestinians' rather unique position under international law may be called into question. Here however, it should be remembered that the statelessness of Palestinians, which is claimed to be a highly politicised and unique situation, is a result of imposed labels and framing by the international community (Fiddian-Qasmiyeh, 2016). Thus, if we put international organisations’ law and discourse aside and consider the cause, perpetuation and consequences of their statelessness, the Palestinians can be seen as being in a very similar situation to many other stateless refugees globally. All stateless situations are political and have their own specificities. This should not be seen as a reason to reject the value of this single group study, but rather a justification for further studies on different stateless refugee populations. In this sense, the findings should be considered as adding to the overall “mosaic” of our understanding (Segal, 1992, p. 93).

## Conclusion

Germany and Sweden, as major destination states in Europe for refugees, have proved to be useful case studies within which to try and unpack the influence of statelessness on asylum destination decision making. The findings of the research show that there was a high degree of destination specificity for the participants. For the majority of the PRS interviewed, this resulted from their desire to reach countries where citizenship could most easily and quickly be acquired, notably Sweden. This was interrelated with other concerns, such as being able to secure a status that would allow the participants to access citizenship, joining family members and limited choice. Economic and educational opportunities or smugglers and agents, played only a minor role in influencing the decision making.

The devaluing of social and human capital, in favour of being able to acquire citizenship as quickly as possible, was striking. Even strong family ties in European states with generous asylum systems and a history of recognising PRS were not sufficient pull factors for most of the participants. The desire to secure a citizenship, by and large, determined their migration trajectory and destination.

Based on the findings, it is argued that if we are to further develop our understanding of the asylum destination decision making of stateless refugees more broadly, then access to citizenship (which includes their ability to claim asylum and if the status they are issued allows them to acquire citizenship) must be considered. With a quarter of all refugees being stateless, it would seem prudent that conceptualisation of refugee movements, as well as policy to manage it, incorporates the possibility that the refugees may be stateless and that their statelessness can be a significant factor in their migration trajectories.

## Abbreviations

ISI: Institute on Statelessness and Inclusion
PRS: Palestinian refugees from Syria
UN: United Nations
UNHCR: The Office of the United Nations High Commissioner for Refugees
UNRWA: The United Nations Relief and Works Agency for Palestine Refugees in the Near East

## Footnotes

1 With regards to the interviews the author undertook, ethical approval was received on the 4/5/2017 from the Regional Ethical Board in Lund.

2 While the impact of the temporary asylum laws introduced in 2016 on the acquisition of Swedish citizenship has yet to be tested, it looks likely that the restricted issuance of permanent residence may pose a significant barrier

## References

[CR1] Archdeacon T (1985). Problems and possibilities in the study of American immigration and ethnic history. International Migration Review.

[CR2] Asylum Information Database. (n.d.). Naturalisation Germany. Retrieved from http://www.asylumineurope.org/reports/country/germany/content-international-protection/status-and-residence/naturalisation.

[CR3] Böcker A, Havinga T (1997). Asylum migration to the European Union: Patterns of origin and destination.

[CR4] Brekke JP, Aarset MF (2009). Why Norway? Understanding asylum destinations.

[CR5] Brochmann G, Seland I (2010). Citizenship policies and ideas of nationhood in Scandinavia. Citizenship Studies.

[CR6] Collyer D (2005). When do social networks fail to explain migration? Accounting for the movement of Algerian asylum-seekers to the UK. Journal of Ethnic and Migration Studies.

[CR7] Darling K (2009). Protection of stateless people in international asylum and refugee law. International Journal of Refugee Law.

[CR8] Day K, White P (2002). Choice or circumstance: The UK as the location of asylum applications by Bosnians and Somali refugees. GeoJournal.

[CR9] DiCicco-Bloom B, Crabtree BF (2006). The qualitative research interview. Medical Education.

[CR10] Doraï MK (2003). Palestinian emigration from Lebanon to northern Europe: Refugees, Networks and Transnational Practices. Refuge.

[CR11] Erekat N (2014). Palestinian refugees and the Syrian uprising: Filling the protection gap during secondary forced displacement. International Journal of Refugee Law.

[CR12] Eshnaiwer, R. (2015). Palestinian Refugees from Syria (PRS) in Jordan: The State of Exclusivism (RSCAS Working Paper, 2015/91). Retrieved from http://cadmus.eui.eu/bitstream/handle/1814/37967/RSCAS_2015_91.pdf?sequence=1&isAllowed=y.

[CR13] Eurostat. (2017a). Asylum Statistics. Retrieved March 4, 2017, from http://ec.europa.eu/eurostat/statisticsexplained/index.php/Asylum_statistics.

[CR14] Eurostat. (2017b). Countries of origin of (non-EU) asylum seekers in the EU-28 Member States 2015 and 2016. Retrieved March 10, 2018, from https://goo.gl/qAnPR5.

[CR15] Eurostat. (2017c). Distribution of First Instance Decisions on (non-EU) Asylum Applications. Retrieved March 10, 2018, from http://ec.europa.eu/eurostat/statistics-explained/index.php/File:Distribution_of_first_instance_decisions_on_(non-EU)_asylum_applications,_2016_(%25)_YB17.png.

[CR16] Eurostat. (2018). Asylum and First-time Asylum Applicants by Citizenship, Age and Sex: Annual Aggregated Data (rounded). Retrieved March, 10, 2018 from http://ec.europa.eu/eurostat/web/products-datasets/-/migr_asyappctza.

[CR17] Feller E, Türk V, Nicholson F (2003). Refugee Protection in International Law: UNHCR’s Global Consultations on International Protection.

[CR18] Fiddian-Qasmiyeh E (2016). On the threshold of statelessness: Palestinian narratives of loss and erasure. Ethnic and Racial Studies.

[CR19] Fripp E (2016). Deprivation of nationality, “the country of his nationality” in article 1A(2) of the refugee convention, and non-recognition under international law. International Journal of Refugee Law.

[CR20] Fullerton M (2005). Comparative perspectives on statelessness and persecution. Kansas Law Review.

[CR21] GLOBALCIT (2017). Global Database on Modes of Acquisition of Citizenship, version 1.0. San Domenico di Fiesole: Global Citizenship Observatory / Robert Schuman Centre for Advanced Studies / European University Institute. Retrieved April 1, 2018, from http://globalcit.eu/acquisition-citizenship/.

[CR22] Goodwin-Gill G, McAdam J (2007). The refugee in international law.

[CR23] Green NL (1994). The comparative method and Poststructural structuralism: New perspectives for migration studies. Journal of American Ethnic History.

[CR24] Hathaway JC (2005). The rights of refugees under international law.

[CR25] Hathaway, J. C., & Foster, M. (2014). *The law of refugee status*, (2nd ed.). Cambridge: Cambridge University Press.

[CR26] Hovil, L. (2016). Ensuring that Today’s refugees are not Tomorrow’s stateless: Solutions in a refugee context. In L. van Waas, & M. Khanna (Eds.), *Solving Statelessness*, (pp. 71–98). Oisterwijk: Wolf Legal Publishers.

[CR27] Human Rights Watch. (2014, August 7). Jordan: Palestinians Escaping Syria Turned Away. Others Vulnerable to Deportation, Living in Fear. Retrieved from https://www.hrw.org/news/2014/08/07/jordan-palestinians-escaping-syria-turned-away.

[CR28] Institute on Statelessness and Inclusion (2014). *The World’s stateless*. Oisterwijk: Wolf Legal Publishers. Retrieved from http://www.institutesi.org/worldsstateless.pdf.

[CR29] Institute on Statelessness and Inclusion & Norwegian Refugee Council. (2016). *Understanding Statelessness in the Syria Refugee Context*. Retrieved from http://www.syrianationality.org/pdf/report.pdf.

[CR30] Joppke C (2010). Citizenship and immigration.

[CR31] Koser, K., & Pinkerton, C. (2002). *The social networks of asylum seekers and the dissemination of information about countries of asylum*. Retrieved from http://www.urbanlab.org/articles/Koser_2002_SocialNetworksOfAsylumSeekers.pdf.

[CR32] Kunz E (1973). The refugee in flight: Kinetic models and forms of displacement. International Migration Review.

[CR33] Manly M, van Waas L (2014). The state of statelessness research: A human rights imperative. Tilburg Law Review.

[CR34] Migrationsverket. (n.d.). *Citizenship for Adults*. Retrieved January 5, 2018, from https://www.migrationsverket.se/English/Private-individuals/Becoming-a-Swedish-citizen/Citizenship-for-adults.html.

[CR35] Neumayer E (2004). Asylum destination choice: What makes some west European countries more attractive than others?. European Union Politics.

[CR36] Palestinians of Syria. (2017, August 26). Action Group for Palestinians of Syria. Retrieved from http://www.actionpal.org.uk/en/post/5714/news-and-reports/palestinians-of-syria-august-26-2017-statistics.

[CR37] Palestinian Refugees: Multiple Displacements and the Issue of Protection. (2017, March). Retrieved from https://reliefweb.int/sites/reliefweb.int/files/resources/al-majdal-59.pdf.

[CR38] Robinson, V. & Segrott, J. (2002). *Understanding the Decision Making of Asylum Seekers* (Home Office Research Study 243). Retrieved from https://www.researchgate.net/publication/248205716_Understanding_the_Decision-Making_of_Asylum-Seekers.

[CR39] Rüegger S, Bohnet H (2015). The ethnicity of refugees (ER): A new dataset for understanding flight patterns. Conflict Management and Peace Science.

[CR40] Sayigh, R. (2013, May 15). The Price of Statelessness: Palestinian Refugees from Syria. *Al-Shabaka: The Palestinian Policy Network*. Retrieved from https://al-shabaka.org/commentaries/the-price-of-statelessness-palestinian-refugees-from-syria/.

[CR41] Segal L (1992). The human face of violence: Hostel dwellers speak. Journal of Southern African Studies.

[CR42] Sen S (1999). Stateless refugees and the right to return: The Bihari refugees of South Asia – Part 1. International Journal of Refugee Law.

[CR43] Sen S (2000). Stateless refugees and the right to return: The Bihari refugees of South Asia – Part 2. International Journal of Refugee Law.

[CR44] Statistics Sweden. (2017). *Asylum-seekers by Country of Citizenship, Sex and Year. Database*. Retrieved March 6, 2018, from http://www.statistikdatabasen.scb.se/pxweb/en/ssd/START__BE__BE0101__BE0101P/Asylsokande/?rxid=85fa76c2-ae70-423c-9fd4-00815291b4ef.

[CR45] The Office of the United Nations High Commissioner for Refugees. (2017). *Figures at a Glance*. Retrieved March 2, 2018 from http://www.unhcr.org/figures-at-a-glance.html.

[CR46] The United Nations Relief and Works Agency for Palestine Refugees in the Near East. (2017a). *Syria Crisis*. Retrieved March 3, 2018, from https://www.unrwa.org/syria-crisis.

[CR47] The United Nations Relief and Works Agency for Palestine Refugees in the Near East. (2017b). *PRS in Lebanon*. Retrieved March 3, 2018, from https://www.unrwa.org/prs-lebanon.

[CR48] The United Nations Relief and Works Agency for Palestine Refugees in the Near East. (2017c). *PRS in Jordan*. Retrieved March 3, 2018, from https://www.unrwa.org/prs-jordan.

[CR49] Tucker, J. (2017). The Indefinite Statelessness of Refugees in Denmark and Sweden: Comparing the Impacts of the Temporary Asylum Laws (MIM Working Paper Series 17:8). Retrieved from https://www.mah.se/upload/Forskningscentrum/MIM/Publications/WPS%2017.8%20-%20Jason%20Tucker.pdf.

[CR50] United Nations General Assembly. (1954). *Convention Relating to the Status of Stateless Persons* (United Nations, Treaty Series, vol. 360, p. 117). Retrieved from http://www.refworld.org/docid/3ae6b3840.html.

[CR51] Vecoli RJ (1972). European Americans: From immigrants to ethnics. International Migration Review.

[CR52] Zolberg AR, Suhrke A, Agyayo S (1989). Escape from Violence. Conflict and the Refugee Crisis in the Developing World.

